# Impact of maternal and neonatal health initiatives on inequity in maternal health care utilization in Bangladesh

**DOI:** 10.1371/journal.pone.0181408

**Published:** 2017-07-25

**Authors:** Mohammad Rifat Haider, Mohammad Masudur Rahman, Md. Moinuddin, Ahmed Ehsanur Rahman, Shakil Ahmed, M. Mahmud Khan

**Affiliations:** 1 Department of Health Services Policy and Management, Arnold School of Public Health, University of South Carolina, Columbia, South Carolina, United States of America; 2 Department of Public Health and Informatics, Jahangirnagar University, Savar, Dhaka, Bangladesh; 3 Maternal and Child Health Division (MCHD), International Centre for Diarrhoeal Disease Research, Bangladesh (icddr,b), Dhaka, Bangladesh; 4 The World Bank, Dhaka, Bangladesh; BRAC, BANGLADESH

## Abstract

**Background:**

Despite remarkable progress in maternal and child health, inequity persists in maternal care utilization in Bangladesh. Government of Bangladesh (GOB) with technical assistance from United Nation Population Fund (UNFPA), United Nation Children’s Fund (UNICEF) and World Health Organization (WHO) started implementing Maternal and Neonatal Health Initiatives in selected districts of Bangladesh (MNHIB) in 2007 with an aim to reduce inequity in healthcare utilization. This study examines the effect of MNHIB on inequity in maternal care utilization.

**Method:**

Two surveys were carried out in four districts in Bangladesh- baseline in 2008 and end-line in 2013. The baseline survey collected data from 13,206 women giving birth in the preceding year and in end-line 7,177 women were interviewed. Inequity in maternal healthcare utilization was calculated pre and post-MNHIB using rich-to-poor ratio and concentration index.

**Results:**

Mean age of respondents were 23.9 and 24.6 years in 2008 and 2013 respectively. Utilization of pregnancy-related care increased for all socioeconomic strata between these two surveys. The concentration indices (CI) for various maternal health service utilization in 2013 were found to be lower than the indices in 2008. However, in comparison to contemporary BDHS data in nearby districts, MNHIB was successful in reducing inequity in receiving ANC from a trained provider (CI: 0.337 and 0.272), institutional delivery (CI: 0.435 in 2008 to 0.362 in 2013), and delivery by skilled personnel (CI: 0.396 and 0.370).

**Conclusions:**

Overall use of maternal health care services increased in post-MNHIB year compared to pre-MNHIB year and inequity in maternal service utilization declined for three indicators out of six considered in the paper. The reductions in CI values for select maternal care indicators imply that the program has been successful not only in improving utilization of maternal health services but also in lowering inequality of service utilization across socioeconomic groups. Maternal health programs, if properly designed and implemented, can improve access, partially overcoming the negative effects of socioeconomic disparities.

## Introduction

Bangladesh shows wide variability in maternal mortality by rural-urban residence, geographical region, and wealth status of households even though the country has achieved remarkable progress in key healthcare indicators like maternal, neonatal, and under-5 mortality in recent years [1]. The annual average rate of reduction of maternal mortality ratio (MMR) over the period of 2000–2015 was quite high but still it was not rapid enough to reach the MMR target within the MDG timeline [2, 3]. The achievements in maternal health were a result of political commitment, effective planning and concerted effort among diverse stakeholders [3]. However, achieving equity in maternal healthcare utilization was not considered explicitly in health-related MDGs and the emphasis was on overall reductions in MMR and other health outcomes rather than lowering disparity in the utilization of services. In fact, in order to achieve a faster rate of progress, it is important to ensure access to essential health care services by all, irrespective of economic status of households [4].

There is also a concern within the health policy decision-makers that maternal-health intervention programs are basically supply-oriented, disregarding the social factors that limit demand for, access to and effective use of maternal health services [5]. Moreover, the behavioral change communication, which is often cited as an effective way of increasing awareness, should incorporate two-way communication between the providers and the users of the services to ensure better quality and utilization [6].

In 2010, the MMR in Bangladesh was 1.12 times in rural areas compared to the ratio in urban areas. The geographic region based disparity was relatively high ranging from MMR of 425 per 100,000 live births in the worst performing region to 168 per 100,000 live births in the best performing region. The richest quintile experienced MMR 37% lower than the national average, whereas it is 20% higher among the poorest quintile [7]. There is also marked disparities in terms of seeking various maternal health care services across different economic strata in the country. Percent of pregnant women seeking ANC from medically trained provider was only 35.6% for the poorest quintile, while it was 90.0% for the richest quintile. Similarly, only 14.9% of poorest quintile mothers delivered in a health facility and 17.9% had a trained provider assisting in the delivery, whereas the percentages were 70.2% and 74.4% for the richest quintile. Only 15.1% of poorest mothers received PNC within 48 hours of delivery by trained personnel while 68.5% of the richest mothers received this care [7]. Care for obstetric complications sought from formal health facility were 14.5% and 46.7% among poorest and richest mothers respectively [8]. Similar inequity persists in all spheres of healthcare service utilization in almost all countries of the world but it is clearly more pronounced in the developing areas. Unless the disparity issues are addressed directly, it will become a major impediment in the achievement of newly stipulated Sustainable Development Goals (SDGs) worldwide [9].

Based on the experiences of the MDG era, United Nations (UN) has set the Sustainable Development Goals (SDGs) for all nations [10, 11]. The SDG 3 goal intends to achieve global MMR target less than 70 per 100,000 live births by the end of 2030 [12]. Underlying theme of SDG 3 is sustainable development achieved through universal coverage of interventions and health care services. Programs should address equity as an overarching objective so that the targets can be achieved at a quicker pace through effective coverage of poorest segment of population. This renewed focus on equity reflects the importance of primary healthcare (PHC) principle adopted in 1978 where equity was the central issue [13]. Therefore, the new initiatives and programs should learn from the experiences of previous successes and failures and one of the lessons is that policy planners should address inequity directly rather than considering it as a passive outcome of improvements in health care delivery system and quality of services.

With an objective of reducing disparities in maternal and neonatal mortality and morbidity, the Government of Bangladesh and United Nations have launched ‘Maternal and Neonatal Health Initiatives in Bangladesh (MNHIB)’ in 2007. The Government of Bangladesh, United Nations Population Fund (UNFPA), United Nations Children’s Fund (UNICEF), and World Health Organization (WHO) have been working together in order to implement the program. The MNHIB was designed to reduce maternal and neonatal mortality and morbidity. The program was implemented in two phases. The initiative intended to ensure that women are aware of their right to safe motherhood and that communities are conscious of how mothers can be assisted in achieving this right. The program adopted a decentralized approach that took into account local needs and demands through participatory local planning processes. District and upazila (sub-district) health and family planning teams developed their own plans and identified principal maternal and neonatal health problems specific to their communities.

Two modes of intervention were implemented in the first phase of the program, e.g., community support system that included planning births involving pregnant mothers and their families and health system mobilization in order to create demand for maternal care services. Community Support System intervention provided by partner Non-Governmental Organizations (NGOs) was adopted in the four program districts (namely, Maulvibazar, Jamalpur, Narail and Thakurgaon). It was hypothesized that birth planning as well as health system mobilization should help enable pregnant mothers and their families to receive a minimum of four ANC visits, safe delivery, PNC and care for obstetric complications from both public and private facilities. The program has also adopted some supply side interventions. The public sector health facilities were strengthened and equipped with required level of human resources, physical capacity, and service level-specific procurement of necessary drugs and equipment. Facilities at all levels were strengthened, from District Hospitals, Maternal and Child Welfare Centers, Upazila Health Complexes, Union Health and Family Welfare Centers and Community Clinics.

One of the most important outcomes of the program was to increase health care utilization, improve access, equity, participation and accountability in maternal and neonatal interventions. This paper is an attempt to understand the impact of this program on inequality reductions in the utilization of maternal healthcare services in the program areas after four years of its implementation. We have used wealth-based inequity measures from the four districts in which the program was implemented to examine changes in equity. Therefore, the results of the analysis should be specific to the program rather than general changes occurring in the country as a whole.

## Methods

The study has been approved by the Institutional Review Board (IRB) of the University of South Carolina. Written informed consent was taken from the eligible mothers before starting the interviews. To measure the impact of MNHI, two cross-sectional surveys were carried out in the four program districts. The baseline survey was conducted in 2008 and the end-line survey was conducted in 2013 after the completion of the first phase. The baseline and end-line surveys collected data from 13,206 and 7,177 women who gave birth in the preceding year. In both surveys stratified cluster sampling was adopted to select the households for interview. Administratively, Bangladesh is divided into 64 districts, which are subdivided into sub-districts called upazila. At the first stage, a sample of villages in rural areas and neighborhoods in urban areas were selected from each of the upazilas. Each upazila, village/neighborhood was selected independently with the Probability Proportion to Size (PPS) of a geographic area (the size was measured in terms of number of households in the area). Then from each cluster, households were selected based on the presence of eligible women (having live birth during last one year preceding the survey).

In both baseline and end-line surveys, data were collected from eligible women from 22 sub-districts of four program districts but the sampling design at end-line yielded a lower sample size. Both surveys used a two-stage sampling procedure although the end-line used 2011 population census rather than 2001 population census as the sampling frame for the selection of villages. Total sample size in the end-line was lower due to funding restrictions.

In both surveys, the interviewers undertook a systematic enumeration of the selected villages by starting from the northwest corner and visiting every household in turn to identify the eligible women. This process continued till the whole enumeration area was covered. The households were then selected randomly from the list of eligible households. If required number of women could not be interviewed from a single village, the interviewer made up the shortfall by visiting as many additional households as required in the neighboring village. Response rate for the baseline survey was 99.2% and 99% in the end-line.

Informed consent was taken from the eligible mothers before starting the interviews and the interviewer asked questions in the presence of another member of the household. The data collection team comprised of both male and female data collectors. If any mother refused to be interviewed by a male interviewer female data collector was assigned to her. Although the sample size of end-line survey was lower than the baseline, the final sample size was still large enough to have the statistical power for the comparison of end-line parameters with those of baseline. Both these surveys were conducted by independent research agencies.

### Statistical analysis

Data from baseline (2008) and end-line (2013) surveys were pooled together and analyzed using statistical package STATA 14.1 [14]. Descriptive statistics were derived to present the socio-demographic characteristics of the sample respondents and their utilization of maternal healthcare services. Age, education and household size of the respondents were collected as continuous variables and were converted into categories for the analysis. Wealth indices were calculated on pooled data using principal component analysis based on housing characteristics and ownership of assets. Households were categorized into five equal wealth classes with 20% of combined sample in each class for the analysis of utilization pattern by socioeconomic status, while continuous wealth score has been used for deriving concentration indices. Antenatal, delivery, postnatal care and care seeking for complications were selected as target indicators for investigating inequality in utilization. Qualified doctor, nurse, paramedic, medical assistant, sub-assistant community medical officer, family welfare visitor, and community skilled birth attendant were considered as medically trained provider for antenatal, delivery and postnatal, and obstetric complications care. Six maternal healthcare indicators were examined: received antenatal care (ANC) at least four times during pregnancy, received ANC from a trained provider, delivery attended by a skilled health provider, delivery at a health facility, received postnatal care (PNC) from a trained provider within 48 hours of the delivery, and received care for obstetric complications at a health facility or from a trained provider, if needed. The information on only last episode of complication during pregnancy and care seeking for that episode was considered for the analysis in both the survey periods. Rich-to-poor ratios were calculated and presented in bar graph over these two time periods to get some preliminary ideas about the extent of inequality in the utilization of maternal and neonatal health services. Statistical analyses of inequality in utilization were performed by calculating the concentration indices for each of the maternal health services and the concentration curves were generated to provide visual representation of changes in inequality by wealth scores from baseline to end-line. Map of the program and comparison districts was prepared with *rgdal* package in statistical software R [15].

### Concentration curve and index

Concentration curve gives a comprehensive view of health inequality by plotting the cumulative percentage of health variable against the cumulative percentage of population ranked from poorest to the richest. The curve also facilitates comparisons of relative inequality between geographic regions and over time. Perfect equality is achieved when the concentration curve coincides with the 45-degree line. If the concentration curve is below or above the equality line, inequality in the utilization of services exists. If the curve lies below the perfect equality line, it implies that the inequality is pro-rich while if the curve is located above the line, it implies pro-poor inequality. The concentration index related to the concentration curve measure the magnitude of inequality and is defined as twice the area between the concentration curve and the line of equality and the index value, by definition, is bounded between -1 to +1. Zero value of the index indicates perfect equality but if the index value gets closer to -1 the disproportionate concentration of health variable among the poor increases and vice-versa [16].

All maternal health indicators used in this study are binary variables and this poses a problem with standard concentration index. Because standard concentration index for these variables may not always be within the [-1, 1] interval [17]. In order to keep the relative inequality variance property of concentration index intact, for bounded variables Wagstaff proposed a modified concentration index by rescaling the standard index [18]. We estimated the corrected concentration index for our study using *conindex* command of STATA [19].

### Comparison with contemporary BDHS survey findings

**Fig 1:**
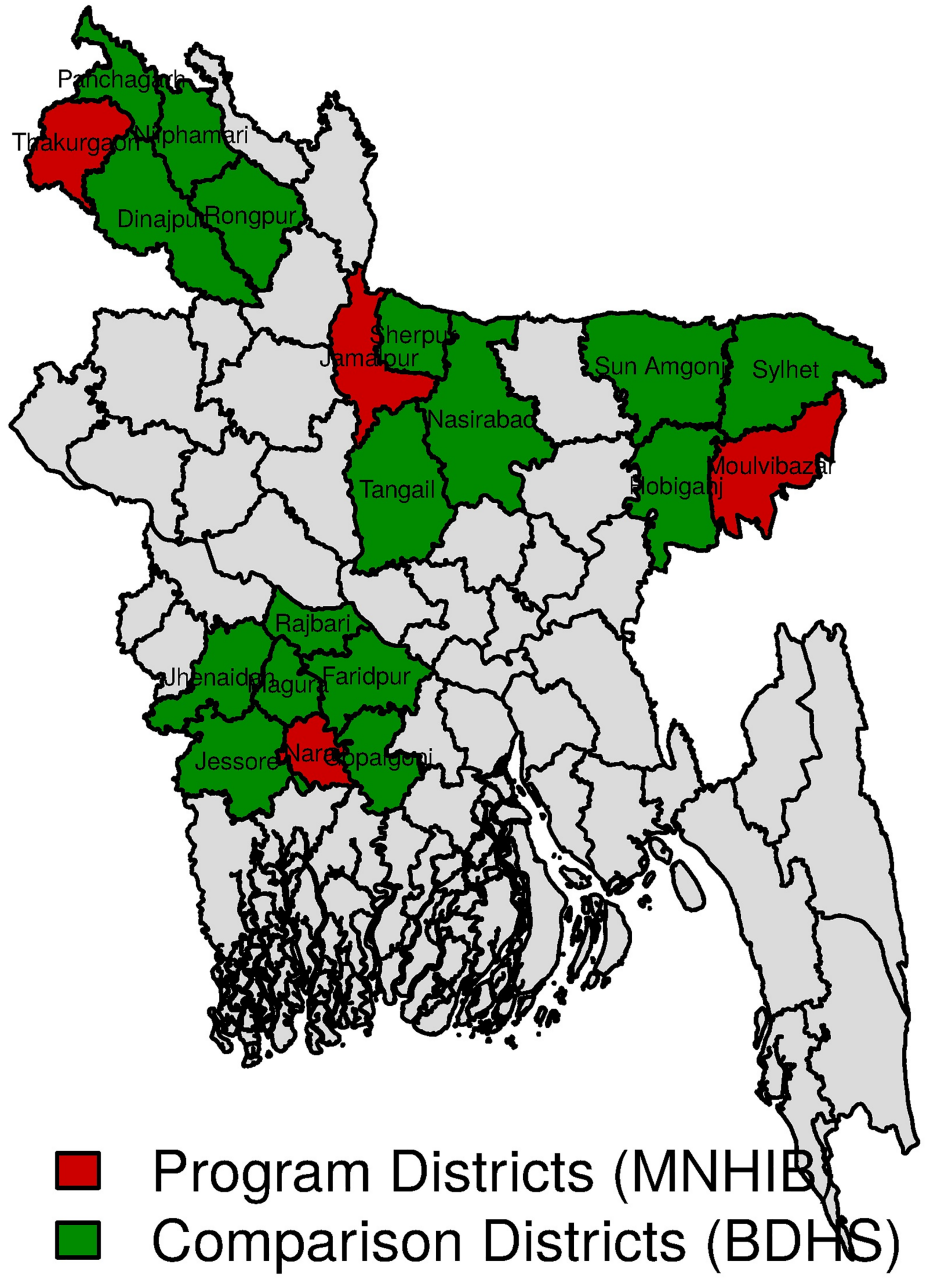
Program districts under MNHIB and comparison districts from BDHS.

Since the program did not collect data from comparison districts either in baseline or in end-line, it was not possible to carry out a difference-in-difference (DID) analysis for estimating the effects of the program. However, a DID analysis has been conducted with contemporary two waves of Bangladesh Demographic and Health Survey (BDHS) conducted in 2007 and 2011. To supplement data collected from the program area, we have looked at the changes in concentration indices between two waves BDHS. Mothers who gave birth in three years preceding the survey were included in the analysis because that makes sure that the time period of the two surveys do not overlap. Comparison districts for each program district were chosen from BDHS data following two criteria: (1) comparison districts should be adjacent, and (2) comparison districts should be in the same division as of the program district (Fig 1). Based on these criteria we selected following comparison districts for each of the four program districts: (1) Thakurgaon: Panchagarh, Nilphamari, Dinajpur, Rangpur; (2) Jamalpur: Sherpur, Mymnensingh, Tangail; (3) Narail: Rajbari, Faridpur, Gopalganj, Magura, Jhenaidah, Jessore; (4) Maulvibazar: Habiganj, Sylhet, Sunamganj. This exercise should be helpful in assessing whether the pattern of changes in inequalities in the program area were similar to changes observed in other nearby districts. Since BDHS also collected information on various maternal health service utilization, we can compare five out of six indicators between the program area and the neighboring areas. For BDHS surveys, we reported the p-value of the differences based on cluster-corrected estimate of variance. In order to examine differences between program and comparison districts, we have conducted a difference-in-difference analysis. Unadjusted odds ratios of the maternal healthcare indicators as well as adjusted odds ratio of the DID estimator are reported. The DID estimator is the coefficient of the interaction term between intervention (1 for program and 0 for comparison) and time (1 for 2013/2011 and 0 for 2008/2007). Since the maternal healthcare utilization indicators are binary variables, the DID estimator shows the difference in end-line from baseline. The linear probability models include following covariates: maternal age, education, location (urban, and rural), division (Chittagong, Dhaka, Khulna, and Rangpur), birth order, wealth quintile (poorest, poorer, middle, richer, and richest).

## Results

### Socio-demographic characteristics

Data from 13,206 recently delivered women in baseline and 7,717 in end-line were included in analysis and their socio-demographic characteristics are reported in Table 1. The distribution of socio-demographic characteristics differs significantly between baseline and end-line. The end-line sample consists of comparatively lower percent of younger women (14.7%) than the baseline (25.2%). Between baseline and end-line there was an increase in the proportion of women with at least primary education or higher and women delivering for the first time (29.7% vs. 33.3%). The proportions of urban population were three folds at end-line than at baseline (7.4 vs. 22.4). Significant improvements were observed in socio-economic status of households between these two periods; proportion of women decreased in lowest wealth quintile (26.6 vs. 8.0) and increased in highest quintile (16.0 vs. 27.4). The average household size changed slightly between these two periods.

**Table 1:** Socio-demographic characteristics of sample respondents by study period.

Characteristics	Baseline (2008) (N = 13,206)	End-line (2013) (N = 7,717)	p-value
	% (n)	% (n)	
**Maternal age (years)**			
15–19 Years	25.2 (3,322)	14.7 (1,057)	0.000
20–29 years	57.6 (7,604)	67.3 (4,826)	
30–49 Years	17.3 (2,280)	18.0 (1,294)	
**Maternal Education (levels of schooling)**			
None	35.5 (4,690)	20.2 (1,448)	0.000
Primary	28.7 (3,796)	29.7 (2,125)	
Secondary	33.1 (4,370)	44.3 (3,169)	
Bachelor or Higher	2.7 (350)	5.8 (414)	
**Birth order**			
First	29.7(3,919)	33.3(2,387)	0.000
Second	28.5(3,768)	29.3(2,106)	
Third	19.4 (2,565)	18.5 (1,328)	
Fourth or higher	22.4 (2,954)	18.9 (1,356)	
**Residence**			
Urban	7.4 (982)	22.4 (1,605)	0.000
Rural	92.6 (12,224)	77.6 (5,572)	
**District**			
Jamalpur	31.9 (4,205)	25.4 (1,821)	0.000
Narail	13.6 (1,800)	11.8 (848)	
Thakurgaon	22.7 (3,002)	29.9 (2,147)	
Moulvibazar	31.8 (4,199)	32.9 (2,361)	
**Wealth Index**			
Poorest	26.6 (3,512)	8.0 (572)	0.000
Poorer	22.4 (2,969)	15.5 (1,111)	
Middle	18.2 (2,399)	26.5 (1,903)	
Richer	16.8 (2,216)	22.6 (1,625)	
Richest	16.0 (2,110)	27.4 (1,966)	
**Household Size**			
1–5 Members	54.1 (7,417)	59.6 (4,277)	0.000
6 or More Members	45.9 (6,059)	40.4 (2,900)	

### Changes in maternal healthcare service utilization

Between baseline and end-line significant improvements were observed in the utilization of maternal and neonatal healthcare services. Receiving antenatal care four times or more from any provider increased from one fifth of pregnant women at baseline to one third at end-line. Antenatal care obtained from trained providers increased from 53% to 61% over these four years. Significant progress has happened in delivery care between these two periods; delivery by skilled providers doubled and institutional delivery more than doubled over the period 2008 to 2013. Visit for health checkup from a trained provider within two days of delivery increased from only 18% at baseline to 32% at the end-line. Seeking treatment for complications from trained providers increased very significantly, from 40% of women needing treatment to 57% (Table 2). Table 2 also shows the difference in use of maternal healthcare services in the comparison areas from the BDHS data. While receiving at least four ANC visits increased from 18% to 30.7%, receiving those from trained personnel decreased from 55.9% to 51%. Other indicators showed increase in utilization over the two survey periods.

**Table 2:** Utilization of maternal healthcare services by study period.

Maternal health indicators	Baseline (2008) (N = 13,206)	End-line (2013) (N = 7,717)	P-value	BDHS (2007) (N = 1,004)	BDHS (2011) (N = 1,346)	p-value
	% (n)	% (n)		% (n)	% (n)	
**Antenatal Care**						
Received ANC at least four times	21.3 (2,807)	32.5 (2,334)	0.000	19.4 (179)	28.5 (384)	0.000
Received ANC from a trained provider	53.2 (7,030)	61.2 (4,392)	0.000	54.4 (503)	48.1 (648)	0.003
**Delivery Care**						
Delivery assisted by skilled health provider	19.4 (2,558)	39.0 (2,608)	0.000	19.1 (191)	24.9 (355)	0.001
Delivered at a health facility	14.8 (1,959)	34.6 (2,311)	0.000	16.6 (166)	28.9 (412)	0.000
**Postnatal Care**						
Received PNC from a trained provider within 48 hours of delivery	18.3 (2,418)	31.5 (2,261)	0.000	18.5 (186)	26.4 (376)	0.000
**Care seeking for complications**						
Number of women had complication any time during antenatal, delivery or postpartum periods	6,651	2,404	-	-	-	-
Sought treatment for obstetric complications from formal health facility/ trained provider	40.2 (2,675)	57.2 (1,374)	0.000	-	-	-

Table 3 shows the program effects on five maternal care utilization indicators using DID analysis adjusting for few socio-demographic and economic variables. The program had some impacts on institutional delivery and delivery by trained personnel while the comparison districts fared better in terms of at least four ANC visits during pregnancy. No differences were found in receiving ANC or PNC within 48 hours of delivery from trained providers between program and comparison areas.

**Table 3:** Program effect on maternal health care utilization: Difference-in-difference analysis.

Maternal health indicators	Unadjusted		Adjusted	
Antenatal Care	DID Estimator (SE)	p-Value	DID Estimator (SE)	p-Value
Received ANC at least four times	0.03 (0.02)	0.175	-0.07 (0.02)	0.001
Received ANC from a trained provider	0.13 (0.03)	0.000	0.05 (0.03)	0.089
**Delivery Care**				
Delivery assisted by skilled health provider	0.16 (0.12 0.20)	0.000	0.12 (0.02)	0.000
Delivered at a health facility	0.09 (0.02)	0.000	0.05 (0.02)	0.005
**Postnatal Care**				
Received PNC from a trained provider within 48 hours of delivery	0.07 (0.02)	0.000	0.03 (0.02)	0.110

### Inequity in maternal healthcare utilization

**Fig 2:**
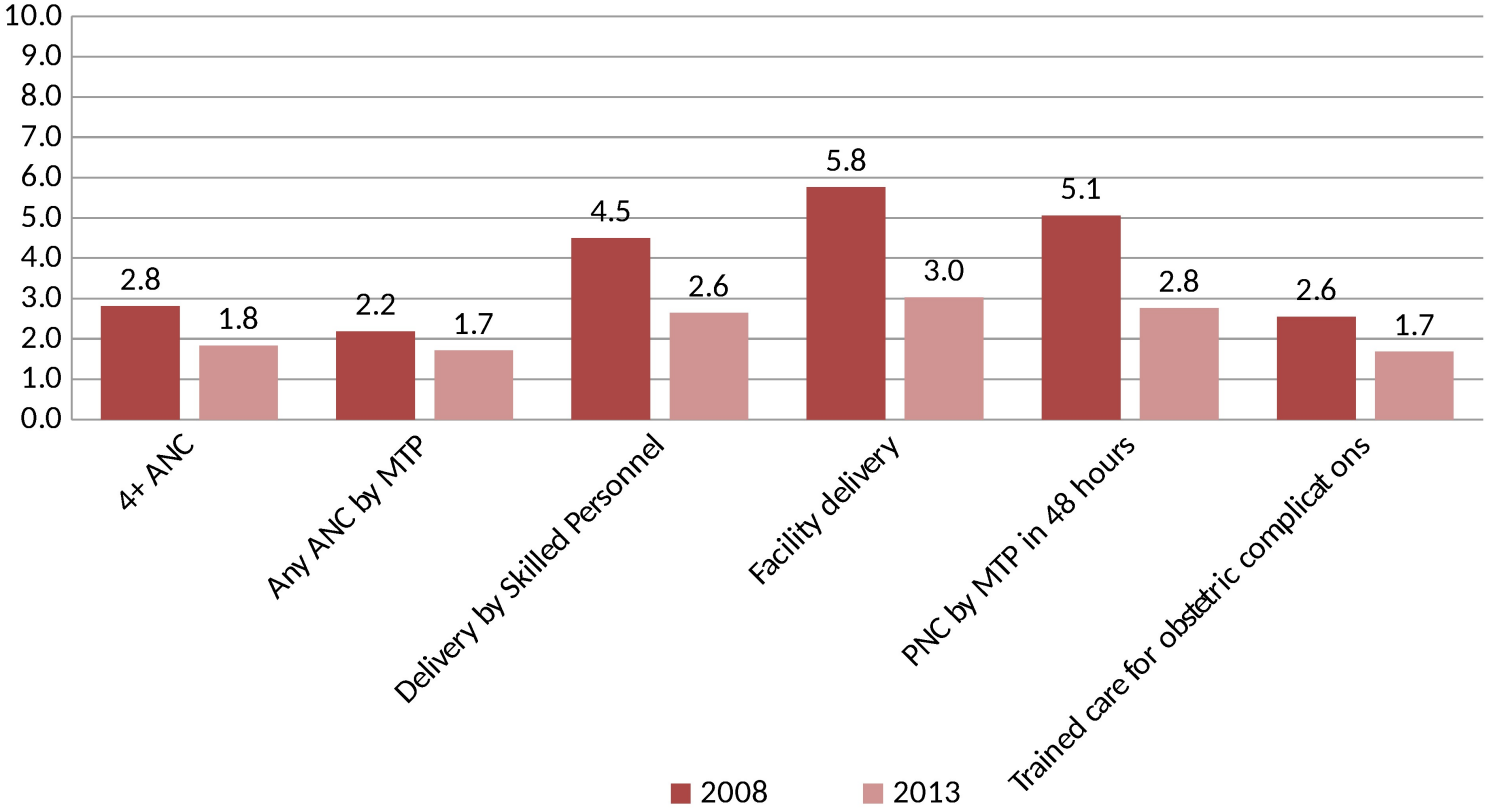
Rich-poor ratio in maternal health indicators by study period.

Fig 2 and Table 4 show that the inequity in utilization of antenatal, delivery, postnatal care and care for complications declined significantly over these two time-periods. Rich-poor ratio of four or more antenatal care visits decreased from 2.8 to 1.8 and the related concentration index reduced from 0.259 at baseline to 0.184 at end-line (p-value <0.001). Similar reduction was also observed for ANC care by medically trained provider; rich-poor ratio decreased from 2.2 to 1.7 and concentration index reduced from 0.337 to 0.272 (p-value <0.001). The reduction in rich-poor ratio was quite large in delivery care utilization indicators; for delivery by skilled birth attendant decreased from 4.5 at baseline to 2.6 at end-line and for facility delivery the ratio declined from 5.8 to 3.0. Inequity in care seeking for obstetric complications from a health facility decreased significantly as well. The series of graphs in Fig 2 shows the concentration curves for 2008 and 2013 for the maternal health service utilization considered in this paper.

**Table 4:** Measure of inequality (Concentration Index and 95% Confidence Interval) by study period.

Indicators	2008			2013			% Change	p-value
	CI^a^	LL^b^	UL^c^	CI	LL	UL		
Received ANC at least four times	0.259	0.247	0.271	0.184	0.169	0.198	-28.98	<0.001
Received ANC from a trained provider	0.337	0.327	0.346	0.272	0.259	0.286	-19.06	<0.001
Delivery assisted by skilled health personnel	0.396	0.384	0.408	0.370	0.356	0.384	-6.61	<0.001
Delivered at a health facility	0.435	0.422	0.449	0.376	0.362	0.390	-13.64	<0.001
Received PNC from a trained provider within 48 hours of delivery	0.419	0.407	0.431	0.332	0.318	0.347	-20.67	<0.001
Sought treatment for obstetric complications at a health facility or qualified provider	0.325	0.312	0.339	0.280	0.257	0.303	-13.95	<0.001

^a^CI = Concentration Index ^b^LL = Lower Limit ^c^UL = Upper Limit

**Fig 3:**
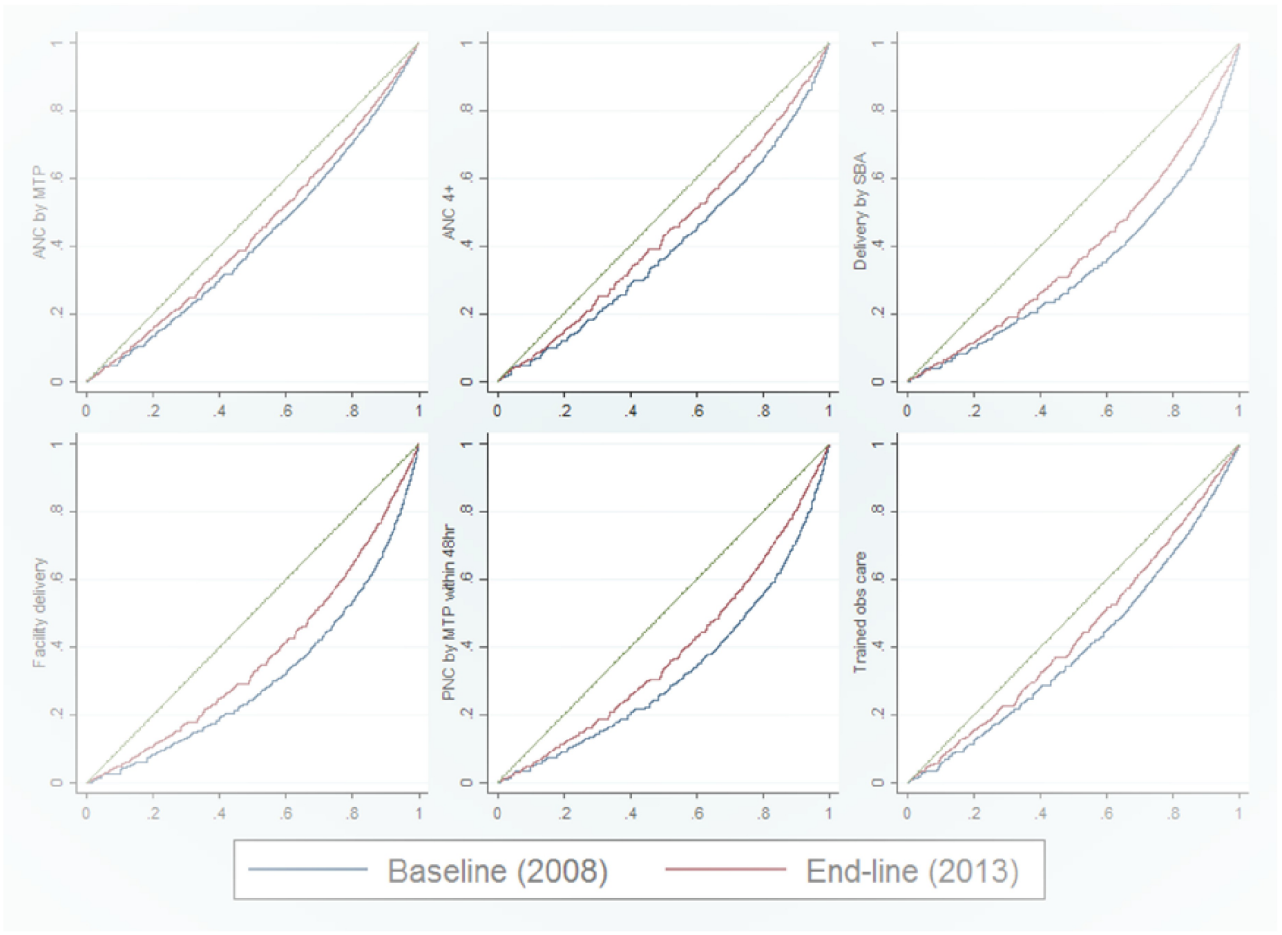
Concentration curves of maternal and neonatal healthcare utilizations by study period.

Table 5 shows the changes in concentration index values for five indicators of maternal health service utilization over two BDHS surveys. Concentration indices decreased significantly for at least four ANC visits (0.506 to 0.345), delivery at a health facility (0.550 to 0.502), and PNC visits by trained personnel within 48 hours of delivery (0.477 to 0.369). However, there are increase in concentration index values for receiving ANC care from trained personnel (0.344 to 0.471) and delivery assisted by trained personnel (0.455 to 0.491). Fig 3 illustrates the concentration curves for each index.

**Table 5:** Measure of inequality (Concentration Index and 95% Confidence Interval) in comparison districts in two BDHS surveys (2007 and 2011).

Indicators	BDHS 2007			BDHS 2011			% Change	p-value	Predicted proportions for 2013*
	CI^a^	LL^b^	UL^c^	CI	LL	UL			
Received ANC at least four times	0.372	0.305	0.439	0.242	0.195	0.290	-34.88	0.114	0.144
Received ANC from a trained provider	0.260	0.215	0.305	0.384	0.351	0.417	47.89	0.026	0.479
Delivery assisted by skilled health personnel	0.368	0.301	0.435	0.468	0.417	0.520	27.16	0.235	0.483
Delivered at a health facility	0.550	0.488	0.628	0.468	0.417	0.519	-14.97	0.826	0.347
Received PNC from a trained provider within 48 hours of delivery	0.376	0.280	0.473	0.414	0.360	0.468	9.98	0.735	0.435

^a^CI = Concentration Index

^b^LL = Lower Limit

^c^UL = Upper Limit

* Based on rate of change of the ratios from 2007 to 2011

Since latest BDHS survey was conducted in 2011 and end-line data from the program area was collected in 2013, for improving comparability of the estimates between the project and the neighboring areas we projected the 2013 level of concentration indices in the comparison area based on the rate of change of the ratios from 2007 to 2011. From the last column of Table 5 we can see that concentration indices for receiving ANC at least four times would have been lower in comparison area than in program area in 2013. On the other hand, concentration indices for receiving ANC from trained personnel, delivery assisted by skilled health personnel, delivery at a health facility, and receiving PNC from a trained provider within 48 hours of delivery would have been higher in the comparison area.

## Discussion

The results show that significant progress was achieved in terms of maternal health care utilization during the study period in the MNHI program districts in Bangladesh. All the indicators of access and utilization of maternal care improved in this area over the years. oUtilization of appropriate type of delivery care doubled over these five years. Proportion of women seeking PNC from trained personnel improved by more than 70%. Although the proportion of women experiencing obstetric complications reduced by about 40%, complication rates are still high (31% of all pregnant women). About 40% of women experiencing obstetric complications did not seek care in 2013 –possibly indicating low confidence of women in the inefficient health system that produces high level of complications.

In our comparative analyses with BDHS data from similar areas indicate that inequality of ANC and delivery services provided by trained personnel and deliveries conducted in health facilities actually improved compared to the improvements observed in neighboring areas. The program areas, however, do not show better improvements in concentration index compared to the concentration indices in the comparison areas for at least four ANC visits and PNC care seeking from trained personnel within 48 hours of delivery. These improvements in the concentration indices can be attributed to the MNHI program which has mobilized both demand side and supply side interventions. On the demand side, the pregnant mothers and their families were educated about the benefits of ANC and PNC visits, delivery at health facility, and the danger signs of pregnancy which includes vaginal bleeding, convulsions/fits, severe headaches with blurred vision, fever and too weak to get out of bed, severe abdominal pain, and fast or difficult breathing [20]. Moreover, community support system intervention also helped to create awareness among community members who, in turn, can help pregnant mothers to reach facilities in time of emergencies. On the supply side, MNHI equipped the facilities with trained personnel and equipment. This enables the health facility to serve the pregnant mothers the required care at different stages of pregnancy and successful treatment in the facilities help to build trust among the people. Poor people are the prime beneficiaries of these interventions because they lack the necessary knowledge, awareness, and community support to reach the hospitals to avail necessary care during pregnancy period. This study results imply that the prevailing high inequity can be reduced following programmatic approaches containing both community awareness and health facility strengthening. It is important to have a proper mix of both demand and supply side interventions because only one intervention, like strengthening facility without any awareness building actually increased inequity rather than decreasing it in one instance in Bangladesh [21].

On the flip side, ANC and PNC visits did not attain the expected result in terms of reducing inequity. It indicates that although both use of four or more ANC visits and PNC visits within 48 hours of delivery by pregnant mother of program areas increased to 32%, these visits are made by more mothers from the richer strata.

Special attention has to be paid to increase ANC and PNC visits among the poorer mother of the society. Pregnancy planning is an integral part of ANC where in the first visit it is recommended to plan for the future complications and delivery to be prepared. Some studies in Cambodia and India have shown that women who attended ANC care are more likely to seek skilled delivery care [22, 23]. The study finding is quite similar with another study where the reason for not seeking ANC services from medically trained personnel (MTP) was due to receiving ANC visits from different healthcare providers like BRAC community health workers. Furthermore, women belonging to the lower socioeconomic groups do not perceive the need for obtaining care from MTP [24]. In the literature, factors found to be negatively associated with seeking ANC from health facilities by poor women were distance to heath facilities, mother’s education and socioeconomic status [25].

PNC visits are also considered as important for proper maternal care because pregnancy related complications can arise after the delivery of the baby. Given the low level of skilled-PNC visits within two days of delivery among Bangladeshi mothers (BDHS, 2014), the program achieved progress in increasing overall PNC visits, but in comparative analysis the rate of reduction in inequity is not up to the mark.

High prevalence of home delivery coupled with low utilization of skilled personnel at delivery has kept the maternal mortality rate relatively high in Bangladesh. Bangladesh adopted the policy of training and deploying skilled birth attendants (SBA) in rural areas, where health facilities are sparse, with a vision to reduce the delays in seeking care during pregnancy. However, use of SBAs was found to be quite inequitable with the poorer section of population not utilizing their services [21, 26]. The MNHIB program has achieved significant progress in moving towards equity in skilled attendance at birth and health facility delivery. We find that Rich-to-poor ratio decreased from 6 to 3 for facility delivery with significant reduction in the Rich-to-poor ratio for skilled attendant at birth. Strengthening health facilities helped to achieve this improvement in skilled attendance. This approach was complemented by the community support system which enabled the pregnant women to reach the facility for delivery or to choose SBA for delivery at home. The improvements observed is similar to the outcomes of the demand side financing (DSF) program in Bangladesh where maternal health voucher scheme increased proportions of deliveries assisted by skilled personnel and/or facility-based delivery for all socioeconomic groups [27].

It is clear from the results that the intervention has improved access to institutional delivery for poor pregnant mothers. A relatively higher proportion of women from poorer groups are also seeking care for obstetric complications. This finding is consistent with the results of a study in rural Bangladesh which found that rural family uses MTP for childbirth when delivery complications are anticipated or encountered [28]. However, improving confidence of the patients in the system requires strengthening of the health system. Provision of appropriate, effective and safe medical treatment improves patient confidence which encourages women, especially the women from poorer sections, to use health care facilities.

Results suggest that equity gap can be addressed even in the context of Bangladesh by adopting both demand and supply side interventions. Behavioral Change Communication (BCC) through community mobilization has positive impact on the pregnant women’s psyche as in many cases even with free of charge of services women are less inclined to take maternal care either from NGO run facility or even government facilities [26]. We all know that behavioral change is not easy and takes time to achieve. But this program has achieved that by creating awareness as well as strengthening the supply side of the health system.

This study is unique in assessing the level of maternal care utilization under the auspices of a program directed to reduce inequity itself. The use of a baseline and end-line data from the program areas provides us with a clear picture of the change in utilization of maternal care over the five years as well as the reduction in inequity thereof. In the absence of any comparison group data we looked at the BDHS data collected at the same time of the baseline (2007) and end-line surveys (2011) and checked the outcome indicators for women reported birth in preceding year residing in the nearby districts of the program districts. We found that those indicators are better in the end-line than the BDHS figure of the contemporary surveys for those nearby districts. Moreover, increase in concentration indices for ANC, delivery and PNC by trained personnel clearly suggests that the program areas has performed better in reducing inequity in at least those two indicators. However, BDHS surveys do not follow a district based sampling frame, rather it follows cluster based sampling method. Therefore, the BDHS samples may not be representative of the district and we should compare with BDHS findings with caution. Though there is a significant difference in urban and rural sample between baseline and end-line, we checked for the concentration indices for urban and rural sample separately and found the progresses in reaching equity in maternal care utilization are similar. Despite conducting these comparative analyses we cannot definitely attribute all these improvements or lack of improvements to the maternal and neonatal program in question. Without a proper comparison group, it is difficult to determine the impacts of a program. However, the fact that both the program area and the adjacent districts showed significant improvements and the pattern of improvements were not similar between these areas imply presence of program effects. The program effects may also have been dampened due to spillover effects of the program in the comparison areas.

Despite considerable progress in reducing equity in three maternal health indicators in the program areas, Bangladesh is still quite far away from achieving universal reproductive health coverage. Even in program areas, 82% women from the poorest quintile and 16% women from the richest are not using skilled attendants at delivery [7]. The interventions being evaluated here were successful in improving access, utilization and equity. Lessons learned from this MNHIB program can be scaled up to strengthen the health facilities as well as to increase demand. The main lesson learned is that through the adoption of appropriate interventions, it is possible to improve utilization of maternal health services as well as socioeconomic equity in service utilization. There is no inherent conflict between increasing access to care and reducing disparity—both can be achieved through the adoption of pro-poor policies and interventions without adversely affecting the health care utilization of relatively better-off in the society.

## Supporting information

S1 File STATA do file. (DO)

S2 File Baseline and end-line appended data set (STATA format). (ZIP)
